# *Albizia amara*: A Potential Plant-Derived Surfactant for Cosmetic and Food Applications

**DOI:** 10.3390/molecules31010081

**Published:** 2025-12-24

**Authors:** Yalini Sadasivam, Valerie J. Pinfield, Anna Trybala

**Affiliations:** Chemical Engineering Department, Loughborough University, Loughborough LE11 3TU, UK; y.sadasivam@lboro.ac.uk (Y.S.); v.pinfield@lboro.ac.uk (V.J.P.)

**Keywords:** *Albizia amara*, *Albizia* spp., saponins, plant-derived surfactants, cosmetics, foods, biosurfactants

## Abstract

Surfactants are essential in cosmetic and food formulations but are still dominated by petrochemical-derived anionic systems associated with irritation, aquatic toxicity and sustainability concerns. Plant-derived saponins offer renewable, biodegradable alternatives, yet only a small subset of saponin-producing species has been developed into commercial ingredients. The genus *Albizia* is chemically diverse and widely used in traditional medicine, with several species empirically employed as cleansers. This review examines *Albizia amara* and related *Albizia* species as prospective sources of plant-derived surfactants for cosmetic and food applications. We summarise ethnobotanical and phytochemical data with emphasis on saponins, flavonoids and macrocyclic alkaloids, and collate the limited quantitative evidence for surface activity, focusing on foaming behaviour, surface tension reduction and shampoo-type formulations, where *A. procera* provides the main interfacial benchmark within the genus. Potential roles of *A. amara*-derived fractions in hair-care products and prospective food systems are discussed alongside current knowledge on toxicity, safety and regulatory constraints. Overall, *A. amara* emerges as a promising but under-characterised saponin source. Priority areas for future work include robust tensiometric characterisation, surfactant-focused extraction and fractionation, systematic formulation studies, and dedicated safety and sustainability assessments to enable evidence-based evaluation against established plant and synthetic surfactants.

## 1. Introduction

Surfactants are indispensable functional ingredients in both cosmetic and food formulations, where they underpin emulsion stability, foaming, wetting, solubilisation and sensory attributes [1,2]. Their widespread use, however, is dominated by petrochemical-derived anionic surfactants such as sodium lauryl sulphate and related homologues, which raise concerns regarding environmental persistence, aquatic toxicity, and skin and mucosal irritation [3]. These issues, together with tightening regulatory and consumer pressure for “milder”, “natural” and more sustainable ingredients, have driven a sustained search for biosurfactant systems that can partially or wholly substitute conventional synthetic agents [4].

Within this landscape, biosurfactants of microbial origin have attracted most attention, but their production often remains constrained by cost, yield, downstream processing, and regulatory hurdles [5]. Plant-derived surfactants, particularly saponins, offer a complementary route that can exploit established agricultural supply chains and ethnobotanical knowledge [6]. Saponins are amphiphilic glycosides capable of lowering surface and interfacial tension, generating stable foam and, in some cases, providing additional bioactivities such as antioxidant, anti-inflammatory or antimicrobial effects [7]. These multifunctional properties align well with current formulation trends in cosmetics and foods, where a single ingredient that combines surface activity with perceived naturalness and added “functional benefits” is highly valued [8].

Although saponins are widely distributed across higher plants, only a limited number of saponin producing species (e.g., *Quillaja saponaria, Sapindus* spp., *Yucca* spp.) are currently used as commercial surfactant ingredients [9], and relatively few plants have been systematically evaluated in modern cosmetic and food formulations. The genus *Albizia* (Fabaceae) is a chemically rich and widely distributed group that includes several species with documented medicinal and household uses. Among these, *Albizia amara* has long been used in South India as a powdered hair wash (“arappu”), either alone or in combination with other botanicals, to cleanse and condition the hair and scalp. This traditional application strongly suggests the presence of surface-active constituents, most plausibly triterpenoid saponins, and provides a real-world use case in which *A. amara* already functions as a natural cleanser.

At the same time, pharmacological and phytochemical studies across *Albizia* species have identified diverse saponins and related secondary metabolites with potential relevance to surface activity, bioactivity and safety [10]. However, these data are scattered across the ethnobotanical, phytochemical and pharmaceutical literature, and have rarely been interpreted through the lens of surfactant science and formulation technology.

The central question, therefore, is not simply whether *Albizia* species contain saponins, but whether *Albizia*-derived saponin systems, particularly from *A. amara*, can meet the performance, safety and regulatory requirements needed for their adoption as functional surfactants in cosmetic and food products. Addressing this question requires an integrated assessment of several dimensions: the phytochemical profile and variability of *Albizia* saponins; the quantitative evidence for surface-active behaviour (surface tension reduction, foaming, emulsification); how these properties compare with established synthetic and plant-derived surfactants [11,12]; and the toxicological and practical formulation constraints that may limit or enable their use.

This review critically evaluates the current state of knowledge on *Albizia* species as potential sources of plant-derived surfactants, with a particular focus on *Albizia amara*. After a concise overview of surfactants, biosurfactants and plant saponins, the discussion concentrates on the ethnobotany and phytochemistry of *Albizia* spp., the evidence for surface-active properties of *A. amara* and related species, and their prospective roles in cosmetic and food formulations. Toxicity, safety and regulatory aspects are examined, and specific research gaps are identified, including the need for robust quantitative surface tension and CMC data, systematic formulation studies, and comprehensive toxicological evaluation. The aim is to clarify whether *A. amara* should be regarded merely as a traditional cleansing plant or as a serious candidate for development into a modern, plant-derived biosurfactant system.

## 2. Surfactants, Biosurfactants and Plant Saponins: A Concise Overview

### 2.1. Surfactants and Biosurfactants in Cosmetics and Foods

Surfactants are amphiphilic molecules that adsorb at interfaces, reduce surface and interfacial tension, and thereby enable wetting, foaming, emulsification and solubilisation in multiphase systems [4,13]. In cosmetics, they underpin cleansing, pigment dispersion, sensorial texture and emulsion stability; in foods, they stabilise foams and emulsions in products such as dressings, beverages, desserts and bakery systems [11]. Industrial practice is still dominated by petrochemical-derived surfactants such as sodium lauryl sulphate (SLS), sodium laureth sulphate, linear alkylbenzene sulfonates and non-ionic ethoxylates, which are inexpensive and well characterised but raise concerns about irritation, aquatic toxicity and incomplete biodegradation [4,6,11].

These concerns, together with regulatory and consumer pressure for “natural” and more sustainable ingredients, have driven interest in surfactants of biological origin [6,13]. Biosurfactants and bio-based surfactants can be defined broadly as surface-active compounds produced by living organisms or derived from renewable feedstocks [4,11]. Microbial biosurfactants (e.g., rhamnolipids, sophorolipids, mannosylerythritol lipids, lipopeptides) combine strong surface activity with high biodegradability and, in some cases, antimicrobial or antiadhesive functions [8,14,15]. They have been investigated as primary or co-surfactants in shampoos, facial cleansers and food emulsions, but wider adoption is constrained by production costs, downstream processing, regulatory hurdles and the need for well-defined composition and safety dossiers [16,17].

Plant-derived surfactants occupy a complementary position, offering sustainability advantages alongside established agricultural supply chains [6,7]. Many plants produce amphiphilic secondary metabolites—most prominently saponins—that can be extracted from biomass already embedded in agricultural value chains [6,18]. In cosmetics, such extracts are used in sulphate-free and “natural” product lines, particularly in cleansers and foam-based formats [11,16]. In foods, selected plant saponins are used or explored as foaming agents and emulsifiers, although regulatory approval and sensory issues (e.g., bitterness, astringency) remain important constraints [7,18]. Overall, current practice points to a gradual diversification away from purely petrochemical surfactants towards portfolios that include microbial and plant-derived systems [11,13], but only a limited number of bio-based surfactants have been characterised in sufficient depth to support broad industrial use. This context underpins the focus of this review on saponin-producing plants, and within that group the genus *Albizia*.

### 2.2. Plant-Derived Saponins as Natural Surfactants

Saponins are glycosides consisting of a hydrophobic aglycone (sapogenin) covalently linked to one or more hydrophilic sugar chains [6,7,19]. The aglycone is most commonly a C_30_ triterpenoid or a C_27_ steroid, while the carbohydrate moiety ranges from a single monosaccharide to branched oligosaccharide chains [18,19]. This combination of a nonpolar core with polar sugar residues gives saponins a clear amphiphilic character: they adsorb at air–water and oil–water interfaces, lower surface and interfacial tension and form aggregates such as micelles above a critical micelle concentration (CMC) [18,20,21].

Structurally, saponins are classified by the nature of the sapogenin (triterpenoid vs. steroidal; oleanane, ursane, dammarane, etc.) and by glycosylation pattern [18,19]. Monodesmosidic saponins carry one sugar chain (typically at C-3), whereas bidesmosidic and tridesmosidic saponins carry two or three sugar chains, often at C-3 and C-28 [7,18,21]. Common sugars include D-glucose, D-galactose, L-arabinose, L-rhamnose, D-xylose, D-fucose and glucuronic acid [18]. This topology determines interfacial orientation and packing: monodesmosidic saponins behave more like “classical” small-molecule surfactants, while multi-desmosidic structures can adopt lay-on configurations that strongly influence interfacial rheology, foam stability and CMC [22,23,24].

The same membrane interaction that underpins surfactant behaviour also explains many biological effects [24,25]. By inserting into lipid bilayers, saponins contribute to plant defence and can cause haemolysis, adjuvant activity and cytotoxicity in animal cells [19,24]. Monodesmosidic triterpenoid saponins are often more haemolytic than bidesmosidic analogues, although activity is highly structure-dependent [24,26]. From a formulation perspective, this dual surfactant–bioactive character is attractive but also constraining: saponins can provide cleansing, antimicrobial and antioxidant functions [7,16], yet require careful toxicological evaluation and concentration control, particularly in oral products and in relation to aquatic toxicity [7,24].

Plant saponins can therefore be regarded as genuine natural surfactants with defined amphiphilic architectures, rather than generic “foaming phytochemicals” [6,18]. However, their structural diversity means that surfactant efficiency and biological activity must be assessed on a species and extract specific basis [7,18,19]. The following subsection places representative plant saponins in a quantitative context relative to a standard synthetic surfactant, providing a benchmark for later discussion of *Albizia* systems.

### 2.3. Performance Snapshot: CMC and Surface-Tension Ranges of Representative Plant Saponins Versus Sodium Lauryl Sulphate

From a formulation standpoint, the key performance metrics for low molecular-weight surfactants are the critical micelle concentration (CMC) and the equilibrium surface tension reached at or near that concentration. Effective surfactants typically reduce the surface tension of water (≈72 mN m^−1^) into the ≈30–40 mN m^−1^ range at practical use levels; higher residual values (≈45–55 mN m^−1^) indicate weaker surface activity but may still be acceptable depending on the application [27]. Plant-derived saponins represent a diverse class of natural surfactants that exhibit considerable variability in their performance characteristics, reflecting differences in their molecular structures and the composition of plant extracts from which they are derived [6].

To place plant-derived saponins in this context, representative CMC and surface-tension data were collated from studies on saponin-rich extracts and purified saponins from a range of species, together with a reference synthetic surfactant, sodium lauryl sulphate (SLS), presented in Table 1. The data illustrate both the potential and variability of plant saponins as practical surfactant candidates. Several systems—notably Acacia concinna, Genipa americana, and Ziziphus joazeiro—reach surface tensions around 30–35 mN m^−1^ under the conditions studied, demonstrating performance comparable to high-efficiency synthetic surfactants [28]. In contrast, other saponin sources remain above 45–50 mN m^−1^ even at relatively high concentrations, indicating weaker surface activity [6]. This variability reflects differences in saponin structure, extraction methodology, purity, and molecular composition across different plant species and plant parts.

The fragmentary nature of the literature is evident in the incomplete datasets presented in Table 1. In many cases, either CMC or full surface-tension isotherms are missing, and some values are reported at single concentrations rather than precisely determined at the CMC. For example, *Camellia oleifera* seed saponins show a surface tension of 50 mN m^−1^ but lack a precise CMC value [29], while *Panax ginseng* and *Quillaja saponaria* have documented CMC values but lack corresponding equilibrium surface tension data [30]. This inconsistency underscores the need for standardised characterisation protocols across saponin research. Triterpene saponins from *Hedera helix* (ivy) species demonstrate the impact of structural modifications on surface activity, with hederacoside C exhibiting higher CMC values due to its increased hydrophilicity compared to hederasaponin B [31].

SLS, included as a benchmark synthetic surfactant, has a reported CMC of 2.0 × 10^−3^ g cm^−3^ and reduces the surface tension of water to approximately 39.2 mN m^−1^ at this concentration [32]. Several plant saponin systems approach or slightly surpass this surface-tension value, though often at higher bulk concentrations. Tea saponin from *Camellia oleifera*, for instance, achieves a CMC of 0.5 g L^−1^ at 30 °C with a minimum surface tension of 39.61 mN m^−1^, demonstrating performance characteristics remarkably similar to SLS [29]. Similarly, *Sapindus mukorossi* (washnut) saponins reach approximately 39 mN m^−1^ at their CMC of 4.50 × 10^−3^ g cm^−3^, placing them in the effective range for practical applications [33]. The most effective systems include *Tamarindus indica*, which achieves 30.02 ± 0.17 mN m^−1^ at 25 °C, and *Genipa americana*, which reaches 31.39 ± 0.15 mN m^−1^, both surpassing SLS in surface tension reduction [34].

However, considerable variability exists within saponin-rich plant materials. *Ilex paraguariensis* (yerba maté) fruit saponins exhibit a substantially higher CMC (1.4946 × 10^−1^ g cm^−3^) and higher residual surface tension (52.8 mN m^−1^), indicating weaker surface activity despite the presence of saponins [6]. Likewise, *Betula pendula* (birch) leaf saponins maintain a surface tension of 45.7 mN m^−1^ at their CMC, approaching the upper acceptable range for some applications [35]. These variations highlight that “saponins” as a broad class are not uniformly equivalent to or superior to synthetic surfactants; rather, their efficacy depends critically on the specific structural composition of individual saponin molecules and the degree of purification of plant extracts.

The data also reveal important relationships between plant part utilised and surfactant performance. Saponins extracted from *Acacia concinna* pods achieve excellent surface tension reduction (≈32.5 mN m^−1^) [33], making them viable candidates for industrial applications in personal care and detergent formulations. In contrast, *Quillaja saponaria* bark saponins, while possessing a very low CMC (2.84 × 10^−4^ g cm^−3^) that indicates strong amphiphilic character, lack published equilibrium surface tension values in many studies, limiting direct comparison [20]. The integration of natural surfactants from *Sapindus mukorossi* and *Acacia concinna* into shampoo formulations has demonstrated that these materials can successfully compete with commercial synthetic formulations while offering enhanced safety profiles [33].

Temperature and pH also influence saponin surface activity. While Table 1 reports data collected at various temperatures (ranging from 20 °C to 25 °C), studies on tea saponins have demonstrated that CMC and surface tension remain relatively stable across moderate temperature ranges (30–60°C), suggesting good thermal stability for practical applications [29]. Furthermore, *Quillaja* and other triterpene saponins exhibit pH-dependent behaviour, with acidic conditions often enhancing surface tension reduction [22].

These comparisons confirm that plant saponins can perform within the range expected of useful surfactants for diverse industrial applications, spanning foods, cosmetics, pharmaceuticals, and household products [6]. However, the data also emphasise critical limitations: the fragmentary nature of published literature, the lack of standardised measurement protocols, and the substantial structural and compositional heterogeneity of saponin-rich plant materials. For the purposes of this review, Table 1 serves primarily as a benchmark summarising what has been achieved for selected saponin systems and highlighting the type of quantitative information (CMC and equilibrium surface tension) that is currently lacking for *Albizia amara* and most other *Albizia* species [36]. Future research should prioritise systematic characterisation of underexplored saponin sources, including standardised extraction and purification methods, to enable more meaningful comparisons with established synthetic surfactants and to unlock the full potential of plant-derived alternatives for sustainable industrial applications.

**Table 1:** Representative CMC and equilibrium surface tension for plant-derived saponins and a reference synthetic surfactant.

Saponin Source	Part Used	CMC (g cm^−3^)	Reduced Surface Tension (mN m^−1^)	Temperature (°C)
*Acacia concinna* [28]	Pods	7.00 × 10^−2^	≈32.5 at 1.00 × 10^−2^ g cm^−3^	20 ± 2
*Agave sisalana* [37]	Leaves	6.84 × 10^−4^	33.57–45.13 at (1.5–4.5) × 10^−4^ g cm^−3^	25
*Albizia procera* [36]	NA	NA	46.6 ± 0.2 mN m^−1^	25
*Bellis perennis* [6]	Flowers	7.60 × 10^−5^	36.8 at 7.60 × 10^−5^ g cm^−3^	20
*Betula pendula* [35]	Leaves	2.4 × 10^−4^	45.7 at 2.4 × 10^−4^ g cm^−3^	20
*Camellia oleifera* [29]	Seeds	NA	50 at 5 × 10^−3^ g cm^−3^	NA
*Equisetum arvense* [6]	Haulm	3.30 × 10^−5^	37.9 at 3.30 × 10^−5^ g cm^−3^	20
*Genipa americana* [34]	Fruits	6.50 × 10^−4^	31.39 ± 0.15 at 6.50 × 10^−4^ g cm^−3^	25 ± 1
*Hedera algeriensis* [31]	Leaves	5.00 × 10^−4^	40 at 5.00 × 10^−4^ g cm^−3^	20
*Ilex paraguariensis* [6]	Fruits	1.4946 × 10^−1^	52.8 at 1.4946 × 10^−1^ g cm^−3^	20 ± 2
*Juglans regia* [6]	Bark	8.80 × 10^−3^	≈45.00 at 1.00 × 10^−2^ g cm^−3^	20 ± 2
*Panax ginseng* [30]	Roots	6.27 × 10^−4^	NA	25
*Quillaja saponaria* [20]	Inner bark	2.84 × 10^−4^	NA	25
*Tamarindus indica* [34]	Fruits	8.70 × 10^−4^	30.02 ± 0.17 at 8.70 × 10^−4^ g cm^−3^	25 ± 1
*Sapindus laurifolia* [34]	Fruits	1.70 × 10^−2^	38.00 at 1.70 × 10^−2^ g cm^−3^	NA
*Sapindus mukorossi* [33]	Pericarp	4.50 × 10^−3^	≈39 at 4.50 × 10^−3^ g cm^−3^	25
*Verbascum densiflorum* [6]	Flowers	3.55 × 10^−4^	41.5 at 3.55 × 10^−4^ g cm^−3^	20
*Zephyranthes carinata* [6]	Bulbs	6.40 × 10^−4^	≈41.25 at 1.00 × 10^−2^ g cm^−3^	20 ± 2
*Ziziphus joazeiro* [34]	Barks	1.064 × 10^−3^	33.94–46.52 at (0.8–5.5) × 10^−4^ g cm^−3^	25
*SLS*	Synthetic	2.004 × 10^−3^	≈39.2	Room temperature

Note: Reduced surface tension refers to the equilibrium surface tension of aqueous solutions containing the indicated saponin or surfactant. Concentration used is the bulk concentration at which surface tension was reported in the original source; in several studies this is at or close to the CMC.

## 3. The Genus *Albizia* as a Source of Saponins

### 3.1. Ethnopharmacology and Traditional Uses

*Albizia* is a pantropical genus within the Fabaceae family comprising over 100 accepted species distributed across Africa, Asia, and the Americas, with substantial representation in tropical and subtropical regions [38]. Ethnopharmacological reviews describe *Albizia* spp. as multipurpose medicinal trees integrated into traditional Indian, Chinese, and African healing systems for treating a diverse range of conditions including respiratory and gastrointestinal disorders, parasitic infections, skin diseases, wound healing, and mood and sleep-related complaints [39]. Among the most prominent medicinal applications, *A. julibrissin* (known as “He Huan Pi” in Chinese medicine) is recognised for its anxiolytic, antidepressant, and sedative properties, typically administered as stem-bark preparations enriched in saponins and flavonoids to address insomnia, anxiety, and depressive states [38]. Similarly, *A. lebbeck* (“siris” in regional nomenclature) remains widely utilised throughout South Asia and parts of Africa as a traditional remedy for asthma, bronchitis, allergic disorders, helminthic infections, and skin diseases [40]. Other species such as *A. adianthifolia*, *A. myriophylla*, and *A. coriaria* appear in regional pharmacopoeias for the management of cough, gastrointestinal disorders, wounds, and infectious diseases [41], underscoring the therapeutic breadth of the genus.

Beyond strictly medicinal applications, several *Albizia* species are historically documented for “detergent” or cleansing functions, reflecting the surfactant properties of their saponin-rich extracts. Bark and pods of *A. lebbeck* have been employed as soap substitutes and in leather tanning processes, their efficacy attributed to the presence of saponins and tannins [34]. In contemporary traditional practice, leaves, bark, or pods of *A. procera* and related species remain incorporated into hair- and body-cleansing formulations in parts of South and Southeast Asia, often in combination with other herbs to enhance cosmetic efficacy and reduce skin irritation [33]. This combination of medicinal and cleansing applications reflects the underlying presence of membrane-active, amphiphilic metabolites—particularly triterpenoid saponins—that are distributed across the genus and capable of functioning simultaneously as therapeutic agents and functional surfactants [6].

### 3.2. Distribution of Triterpenoid Saponins and Other Metabolites in Albizia

Comprehensive phytochemical reviews of the genus establish that many investigated *Albizia* species are rich in triterpenoid saponins, which represent a major class of isolated constituents alongside flavonoids, phenolic glycosides, and lignans [42]. A broader metabolomic survey examining nine *Albizia* species—including *A. lebbeck*, *A. julibrissin*, *A. odoratissima*, *A. procera*, *A. anthelmintica*, *A. guachapele*, *A. myriophylla*, *A. richardiana*, and *A. lucidior*—resulted in the tentative identification of 64 metabolites, predominantly comprising flavonoids, phenolic acids, saponins, and alkaloids [38]. This genus-level context establishes the chemical rationale for classifying *A. amara* as a saponin-containing species and provides a foundation for understanding the likely structures and bioactivities of its constituent metabolites.

At the level of individual molecular structures, multiple *Albizia* congeners including *A. lebbeck*, *A. chinensis*, *A. julibrissin*, *A. gummifera*, *A. adianthifolia*, and *A. myriophylla* have yielded well-characterised oleanane-type and related triterpenoid saponins [39]. Although the specific aglycones and sugar chains differ between species, reflecting evolutionary and biosynthetic divergence, a consistent structural pattern emerges across the genus: most characterised *Albizia* saponins are based on pentacyclic triterpenoid cores—commonly derived from oleanolic acid, echinocystic acid, or acacic acid—bearing two or more sugar residues composed from a relatively narrow set of monosaccharides including arabinose, rhamnose, xylose, glucose, and occasionally N-acetyl-hexosamines [38]. These structurally diverse yet fundamentally related saponins frequently display pronounced membrane-active properties including hemolytic activity and in vitro cytotoxicity, reflecting their amphiphilic character and capacity to disrupt lipid bilayers [27].

In addition to saponins, comprehensive phytochemical screening of *Albizia* species has documented the presence of an array of flavonoid glycosides (e.g., quercetin, kaempferol, and myricetin derivatives), phenolic glycosides, lignans, and fixed oils [38]. These secondary metabolite classes contribute complementary antioxidant, anti-inflammatory, antimicrobial, and other bioactivities of substantial relevance to potential cosmeceutical and nutraceutical applications [39]. Analysis of *A. procera* leaf extracts, for example, revealed the presence of saponins, steroids, tannins, glycosides, and flavonoids, with the ethyl acetate fraction demonstrating antioxidant activity comparable to standard ascorbic acid [36]. The stem bark of *A. coriaria* has yielded six bioactive compounds including lupeol, lupenone, betulinic acid, acacic lactone, catechin, and benzyl alcohol, compounds recognised in the literature for their diverse biological activities [41]. The coexistence of multiple bioactive metabolite classes within individual *Albizia* species suggests that crude plant extracts may exhibit synergistic or antagonistic interactions, potentially modulating both therapeutic efficacy and safety when formulated for pharmaceutical or cosmeceutical use.

Collectively, genus-wide evidence supports the conclusion that triterpenoid saponins form a recurrent chemical theme throughout *Albizia*, coexisting with flavonoids and other bioactive metabolite classes whose presence may substantially modulate both the efficacy and safety of crude extracts employed therapeutically or in cosmetic formulations [6]. This metabolic complexity underscores the necessity for comprehensive chemical characterisation of individual *Albizia* species before confident prediction of their functional properties or therapeutic profiles can be made.

### 3.3. Cleansing and Surface-Active Properties Within the Genus

The saponin-rich chemical profile that underpins the broad pharmacological activities documented across *Albizia* also provides the molecular basis for the traditional cleansing and detergent uses of multiple species within the genus. Ethnobotanical and phytopharmacological reviews document that bark and pods of *A. lebbeck* have been employed for generations as soap or detergent substitutes in traditional cleaning practices, while several species—including *A. lebbeck*, *A. procera*, and *A. julibrissin*—continue to be incorporated into hair- and body-cleansing formulations, typically in combination with other plant materials to optimise both cleansing efficacy and skin compatibility [34].

The physicochemical basis for these cleansing applications has been systematically examined in a limited number of studies characterising the surface-active behaviour of *Albizia* saponin extracts in aqueous systems. Studies evaluating natural surfactants extracted from *A. concinna* and *A. procera* documented their capacity to reduce surface tension, generate stable foam, and provide measurable detergency properties when tested against commercial baby shampoo formulations [33]. These investigations demonstrated that *A. procera* extracts successfully reduce surface tension, generate foam, and exhibit distinct foaming and emulsifying characteristics comparable to low-performance synthetic detergents [34]. Additionally, crude *A. procera* saponin extracts have been documented to exhibit moderate surface tension reduction, maintain low alkalinity (desirable for skin and hair compatibility), and display clear foaming and emulsifying properties with changes in viscosity and electrical conductivity consistent with micelle formation in aqueous solution [36].

However, systematic quantitative characterisation remains limited for most *Albizia* species. The available data indicate that *Albizia* saponin extracts exhibit surfactant-like behaviour in aqueous solutions and can be effectively formulated into simple shampoo-type products [34]. Nevertheless, as discussed in detail in Section 5, the surface tension performance of *Albizia* saponins is generally less pronounced than that of benchmark synthetic surfactants such as sodium lauryl sulphate and the highest-performing plant saponin alternatives [6]. This performance gap reflects both the compositional heterogeneity of crude plant extracts and, in many cases, lower intrinsic amphiphilicity compared to specifically engineered synthetic molecules.

At present, most *Albizia* species, including *A. amara*, remain only partially characterised from a surfactant-science perspective. While traditional cleansing uses are reasonably well documented through ethnobotanical records and qualitative foam tests have been conducted on several species, systematic interfacial measurements—including surface tension isotherms, precise CMC determinations, foam stability assays, and emulsion stability data—are available for only a small subset of species within the genus [38]. *A. procera* and *A. lebbeck* represent the most extensively characterised species from a surfactant standpoint, yet even for these species comprehensive datasets remain fragmented and sometimes contradictory across different studies. This genus-level context frames the more detailed discussion in Section 4 and Section 5: *A. amara* belongs to a demonstrably saponin-rich, surface-active genus [38], yet its own saponin structures and surfactant properties remain poorly defined and insufficiently characterised compared with congeners such as *A. procera*, *A. lebbeck*, and *A. julibrissin*. Comprehensive characterisation of *A. amara*—including targeted phytochemical profiling, quantitative saponin determination, and systematic surfactant performance evaluation—would provide essential context for understanding its potential applications in cosmetic and household cleaning formulations while also clarifying how its properties relate to the broader chemical and functional diversity documented across the genus.

## 4. Phytochemistry of *Albizia amara* and Related *Albizia* Species

### 4.1. A. amara Botany and Distribution

*Albizia amara* (Roxb.) Boiv. is a small to moderate-sized, much-branched deciduous tree in the family Fabaceae, subfamily Mimosoideae [43,44], with smooth to scaly dark-green bark, a rounded crown and bipinnate leaves bearing numerous small linear leaflets. Trees within the Fabaceae family, including *Albizia* species, are portrayed as pioneer species in deciduous or monsoon woodland and savanna scrub vegetation [43], characterising their ecological importance across tropical regions. Floristic and agroforestry sources place *A. amara*’s native range across tropical and subtropical Africa (from Eritrea and Sudan southwards to South Africa, including Botswana, Ethiopia, Kenya, Tanzania, Zambia and Zimbabwe) and into the Indian subcontinent, particularly dry regions of India and Sri Lanka (Deccan Plateau, Eastern and Western Ghats) [45,46]. Within these regions, the species is characteristic of dry deciduous woodland and bushland environments [43] and possesses the ability to colonise degraded or abandoned agricultural land [45]. This ecological adaptability reflects its value in agroforestry and land restoration programmes across its native range.

Traditional uses of *A. amara* are diverse and deeply embedded in indigenous medical systems [47]. Decoctions or powders of leaves, bark and seeds are reported in ethnomedicinal practice for treatment of diarrhoea, skin diseases, gonorrhoea, piles, poisonous bites and respiratory complaints [39,45]. Seeds are often described as astringent, while leaves and bark are applied topically to ulcers, wounds and inflammatory skin conditions [45]. In South India, a particularly significant traditional use involves processing leaves into “arappu” hair-wash powder by shade-drying and milling [34]. The powder is used as a shampoo and conditioner, frequently in combination with other saponin-rich plants such as *Acacia concinna* and *Sapindus* spp. [34]. This detergent-like traditional use is consistent with the presence of amphiphilic constituents, particularly saponins, in the foliage [34]. Such traditional applications are notably supported by the documented use of plant-based biosurfactants and natural saponins in cosmetic formulations [32,48], demonstrating the scientific basis for the plant’s long-standing cosmetic applications [16].

### 4.2. Phytochemical Profile of Albizia amara

Several phytochemical surveys demonstrate that *A. amara* contains a complex mixture of secondary metabolites rather than being dominated by a single chemical class [39,45]. Qualitative screening of leaf, bark, root and seed extracts using classical colour reactions and chromatographic methods typically reports saponins, terpenoids/triterpenoids, phenolics, flavonoids, tannins, alkaloids, glycosides and sterols [45,49]. This chemical complexity is characteristic of many medicinal plants in traditional systems, where multiple compound classes work synergistically to produce therapeutic effects [49].

For leaves specifically, multiple independent studies agree on the presence of saponins together with terpenoids, flavonoids, tannins and alkaloids [38]. Preliminary phytochemical screening of 50% ethanolic leaf extracts gave positive tests for flavonoids, tannins, terpenoids, phenols and saponins [36]. Gas chromatography-mass spectrometry (GC–MS) analysis of ethanolic leaf extracts has identified a range of low- and medium polarity constituents such as long-chain hydrocarbons, fatty acid derivatives and terpenoid-type molecules [38]. Furthermore, Fourier transform infrared (FTIR) spectroscopy shows characteristic absorption bands assignable to alcohols, phenols, alkanes, amines, nitro groups and alkenes [38,50]. These spectroscopic data collectively confirm that crude leaf extracts contain both hydrophobic and hydrophilic functionalities [36], consistent with the presence of multiple amphiphilic constituents when such extracts are incorporated into formulations. This dual polarity is particularly important for understanding the plant’s traditional use as a natural detergent, as amphiphilic molecules are essential for surfactant activity [16].

Phenolic and flavonoid contents have been quantified in different solvent fractions of *A. amara* leaves and seeds. One comparative study on leaves and seeds reported total phenolics in the approximate range of 6–12 µg gallic acid equivalents mL^−1^ and total flavonoids around 11–13 µg catechin equivalents mL^−1^ [38], with methanolic seed fractions showing particularly high values and associated in vitro antioxidant activity. HPTLC (high-performance thin-layer chromatography) and HPLC-based profiling have further confirmed flavonoids as major leaf constituents and correlated these with strong free radical scavenging activity in DPPH (2,2-diphenyl-1-picrylhydrazyl) and related assays [38,51]. More detailed structural work has led to the isolation of myricitrin and related flavonol glycosides from leaves, as well as a flavanone (5-methoxy-3′,4′,5′-trimethylflavanone) from Sudanese *A. amara* foliage [39]. These isolated compounds have been linked to specific biological effects in rodent models [39]. Collectively, these studies establish flavonoids and flavanones as well-characterised polar constituents of *A. amara* foliage with demonstrated antioxidant capacity [36,38].

Seeds of *A. amara* have been the primary source of macrocyclic spermine alkaloids known as budmunchiamines [38]. Using a DNA-interaction-based HPLC screening system, researchers isolated several macrocyclic pithecolobine alkaloids (budmunchiamines A–C) from seed extracts and demonstrated that they interact strongly with calf thymus DNA and are cytotoxic to multiple mammalian cell lines in vitro [38]. Subsequent work by other researchers identified additional analogues (budmunchiamines D–I) from *A. amara* seeds, again using DNA interaction as the guiding assay [38]. A comprehensive review of spermidine/spermine alkaloids reiterates that budmunchiamine-rich *A. amara* seed extracts exhibit pronounced cytotoxic, antimicrobial and enzyme-inhibitory activities [38]. These macrocyclic alkaloids are not surfactants in the conventional sense [38], but their high bioactivity is directly relevant to safety and regulatory considerations when whole-plant materials (particularly seeds or mixed powders) are used in topical or ingestible products. Such considerations are important in the context of traditional use, where multiple plant parts may be combined in preparations [52].

With respect to saponins in *A. amara*, the evidence is robust at the level of chemical class but weak at the level of fully elucidated individual structures [38]. Several phytochemical and pharmacological reviews on *A. amara* explicitly list triterpenoid saponins among its major constituents, alongside phenols, flavonoid glycosides and tannins [39,45]. Foam tests and generic “saponin-positive” reactions in leaf and bark extracts support this classification [38], and some authors have suggested that triterpene saponins may contribute to reported hepatoprotective, antihyperlipidaemic and antioxidant effects of *A. amara* extracts [39]. Indeed, saponins as a class are known to exhibit a wide variety of biological activities including surface-active properties [7,53]. However, in contrast to several other *Albizia* species, no *A. amara* saponins with fully defined aglycone and sugar-chain structures have yet been reported in the primary literature [38]. Structural work has focused much more extensively on flavonoids (e.g., myricitrin, flavanones) and macrocyclic alkaloids (budmunchiamines) than on saponins [38].

Recent GC–MS- and chromatographic-based studies, including FTIR/HPTLC/GC–MS fingerprints and fractionation of leaf and bark extracts for antioxidant and antimicrobial testing, confirm that *A. amara* contains mixtures of polar and moderately non-polar constituents with significant radical-scavenging and antimicrobial activity [38,54]. However, these studies again stop short of isolating and fully assigning individual saponins [54]. This represents a clear research gap: *A. amara* is widely treated in the literature as a “saponin-containing” species and is used practically as a cleanser in traditional cosmetic applications [34], yet its saponin structures remain largely inferred from genus-level patterns rather than experimentally defined through primary structural characterisation [38]. The absence of detailed saponin structure data contrasts markedly with the well-documented saponin chemistry of related *Albizia* species [38], highlighting an important opportunity for future phytochemical research focused on the complete structural elucidation of this bioactive compound class in *A. amara*.

### 4.3. Insights from Other Albizia Saponins Relevant to A. amara

Since *A. amara* itself has not yet yielded fully characterised saponins, structure–activity expectations must be inferred from better studied *Albizia* congeners. Reviews of *Albizia* phytochemistry emphasise that triterpenoid saponins are major active components across the genus [43,55], with many species yielding structurally diverse oleanane-type and related triterpenoid glycosides [56,57]. This chemical complexity highlights the genus’s potential as a source of bioactive amphiphilic molecules.

In *A. chinensis*, three new oleanane-type triterpene saponins (albizosides A–C) were isolated from stem bark [58]; their structures feature echinocystic or oleanolic acid cores glycosylated with multi-sugar chains. These compounds exhibit cytotoxicity against several human tumour cell lines and haemolytic activity against rabbit erythrocytes [59]. Two further oleanane-type saponins, albizosides D and E, together with the known julibroside J8, have also been reported from *A. chinensis* and show moderate cytotoxic activity [60]. In *A. grandibracteata*, three oleanane-type triterpenoid saponins (grandibracteosides A–C) were isolated from leaf extracts and structurally elucidated by NMR and MS [61]; these compounds showed significant inhibitory activity against KB and MCF-7 tumour cell lines in vitro. These findings from related species establish a clear pattern of anticancer activity associated with oleanane-type saponins within the genus.

Other *Albizia* species further reinforce this structural and bioactivity theme [62]. *A. julibrissin* has yielded numerous julibrosides—complex triterpenoid saponins with multi-sugar chains—associated with anti-tumour, anti-angiogenic and immunomodulatory properties [61]. Meanwhile, *A. gummifera*, A. subdimidiata and A. coriaria have produced additional oleanane-type and anthranilate-modified saponins with antitrypanosomal, anti-cancer or other bioactivities [63]. The structural characterisation of pentacyclic triterpene glycosides from related plant families has revealed that compounds featuring echinocystic acid, oleanolic acid, and related cores display both antimicrobial and cytotoxic properties [56,64]. Across these species, the saponins typically share three key structural features: (i) pentacyclic triterpenoid aglycones, most commonly oleanolic-, echinocystic- or acacic-acid related cores [59,65]; (ii) two or more sugar residues, often comprising arabinose, xylose, rhamnose, glucose and occasionally N-acetyl-hexosamines [24,65]; and (iii) pronounced membrane-active properties, including haemolysis, cytotoxicity and adjuvant effects [66,67], which together indicate strong interaction with lipid bilayers [68].

From a surfactant-science perspective, these architectures are structurally consistent with those of potent biosurfactants and plant-derived surface-active compounds [20,69,70]: a bulky but amphiphilic triterpenoid “tail” linked to one or more highly hydrophilic sugar chains, expected to support interfacial adsorption, micellisation and foam formation [22,71]. The detailed study of saponin-membrane interactions has demonstrated that both the aglycone portion and sugar chain composition determine surface activity [65]. Natural plant-derived saponins from species including *Sapindus mukorossi*, *Quillaja saponaria*, and related genera have been extensively characterised as natural surfactants with excellent foaming and emulsifying properties [7,72]. At the same time, the strong membrane activity observed for several *Albizia* saponins—especially monodesmosidic oleanane derivatives [67]—suggests that safety margins for cosmetic and oral applications may be narrow if such compounds are present at high levels, particularly in crude extracts [73,74].

In the specific case of *A. amara*, genus-level evidence therefore suggests that its uncharacterised saponin fraction may contain oleanane/echinocystic-type triterpenoid glycosides analogous to those reported from *A. chinensis* [62], *A. grandibracteata* and *A. procera*. This inference is consistent with generic references to “triterpene saponins” in *A. amara* reviews [62] and with its observed foaming and detergent properties in traditional and experimental hair-cleansing preparations [34]. However, without direct isolation and structural characterisation from *A. amara* itself, such analogies remain tentative [62].

For this review, two implications follow. First, the presence of triterpene saponins in *A. amara* is reasonably well supported at the class level [43], and experience from related *Albizia* species demonstrates that triterpenoid saponins in this genus can display marked membrane activity [68] and clear surfactant and emulsifying behaviour [6,20]. Comparative studies of plant-derived saponins confirm their utility as natural emulsifiers with excellent surface-active properties [69,70]. Second, in contrast to *A. chinensis*, *A. procera* or *A. julibrissin*, *A. amara* still lacks a defined saponin “fingerprint” (identities, relative abundances and structure–activity relationships) [62]. Given the presence of highly bioactive budmunchiamine alkaloids and the cytotoxic/haemolytic profile of saponins in congeners, careful isolation and characterisation of *A. amara* saponins is a prerequisite for evidence-based evaluation of its suitability as a biosurfactant source in cosmetic and food formulations [62]. This targeted phytochemical work would address a critical knowledge gap and enable comprehensive risk-benefit assessment for product development. The subsequent sections therefore focus on available performance data (surface tension, emulsification, bioactivity) and highlight where targeted phytochemical work is most urgently needed.

## 5. Surface-Active Properties of *Albizia*-Derived Saponins

The quantitative evidence for surfactant behaviour in the genus *Albizia* remains limited and unevenly distributed across species. Most of the “hard” interfacial data concern *A. procera* leaf or pod extracts, whereas for *A. amara* the record is dominated by ethnocosmetic use and simple foaming tests rather than full tensiometric characterisation. In this section, we first summarise qualitative and formulation-level observations, then review the available quantitative data, and finally position *Albizia* saponins within the broader plant-saponin performance landscape.

Very recent work has begun to address this by quantifying the apparent surface tension and emulsifying behaviour of raw *A. amara* leaf powder suspensions in water, providing baseline physicochemical data for minimally processed material [75].

### 5.1. Qualitative Foaming, Emulsifying and Detergency Evidence

Ethnobotanical and ethnopharmacological sources describe *A. amara* leaf powder (“arappu”) as a traditional hair and body cleanser in parts of South India, used either alone or in combination with other saponin-rich plants such as *Acacia concinna* (shi-kakai) and *Sapindus* spp. (reetha/soapnut) [34]. In these reports, *A. amara* leaf preparations are associated with removal of oil and dirt from the scalp and are sometimes cited for adjunct benefits such as reducing dandruff and hair fall, in line with its recognised medicinal use for skin conditions [39]. Although these descriptions do not include formal surfactant measurements, they are consistent with the presence of triterpenoid saponins in the leaves and provide contextual evidence that *A. amara* material can support foaming and detergency in everyday use.

The most explicit examination of *A. amara* as a hair-cleansing agent is the poly-herbal shampoo-powder study by Rao, who screened 21 herbs traditionally used in hair care. Under the extraction and assay conditions employed, *A. amara* leaves showed the highest crude saponin yield (0.77 g in the tested extract) and a foaming index >100 across ten serial dilutions in water, indicating persistent foam formation [76]. Powder shampoos formulated with *A. amara* as one of several plant ingredients exhibited slightly acidic pH, visually stable foam in cylinder tests, and satisfactory cleansing and sensory performance in small-scale panel evaluations [76]. Because these formulations are multi-component systems, the specific contribution of *A. amara* cannot be isolated; nonetheless, the data show that *A. amara* leaf material can be incorporated into shampoo-like powders that meet basic hair-cleansing criteria without synthetic surfactants in the solid phase [77].

Genus-level reviews note that other *Albizia* species, including *A. lebbeck* and *A. procera*, have also been used traditionally as cleansing agents for skin and hair [43], alongside more widely studied applications such as anti-inflammatory and antiparasitic treatments. In most cases these plant parts (bark, leaves, pods) are used in mixtures with other herbs, so the specific surfactant contribution of *Albizia* saponins cannot be quantified from the ethnobotanical literature alone. Qualitative and formulation reports therefore support the conclusion that *A. amara* and related *Albizia* species can provide foaming and cleansing sufficient for traditional and herbal hair-care preparations [76], but they do not yet provide quantitative surfactant metrics necessary for rigorous comparison with established natural or synthetic alternatives.

To contextualise the *Albizia* data within the broader plant-saponin landscape, recent comprehensive studies have established quantitative benchmarks for plant-derived saponins in shampoo formulations and surface-active applications. Plant-based saponins from *Sapindus mukorossi*, *Quillaja saponaria*, and *Camellia oleifera* have been characterised with detailed tensiometric and foaming measurements [29,32]. For example, saponin extracts from *C. oleifera* and tea saponins have demonstrated CMCs in the range of 0.05–0.5 g L^−1^, with corresponding surface tension reductions from 72 mN m^−1^ to approximately 30–40 mN m^−1^ at or above the CMC [29,78]. Tea saponins specifically showed foam volumes reaching 490 mL with half-lives of 2350 s, indicating excellent foam stability [29]. The foaming ability of *C. oleifera* saponins was reported as 37.1% relative to 0.5% SDS solution, with an R_5_ foam stability value of 86.0%, demonstrating moderate but consistent foam retention [79].

In shampoo formulations specifically, novel green shampoos formulated with plant-derived saponins and microbial biosurfactants achieved surface tensions of 31.73–38.83 mN m^−1^, detergency values of 57.74–64.45%, and foam volumes with stability maintained even after 60 min [32]. These formulations performed comparably to commercial synthetic shampoos in terms of pH, wetting time, and foam stability [32]. Additionally, studies on herbal shampoo formulations combining multiple plant sources (including Shikakai, Reetha, and Amla) demonstrated the feasibility of achieving acceptable physical and chemical properties without synthetic surfactants [76].

For *Albizia procera*, the most studied species within the genus with respect to surfactant properties, surface activity assessments confirmed the plant’s excellent surface-active characteristics, showing good washing power and dirt dispersion that was equivalent to commercial detergents [80]. The extract demonstrated good salt and hard-water resistance, properties that are critical for practical hair-care applications [80]. These findings suggest that *Albizia* species possess the fundamental properties necessary for functioning as natural surfactants, even if precise quantitative characterisation for *A. amara* specifically remains incomplete.

### 5.2. Quantitative Surface Tension and CMC Data for Albizia Saponins

To date, there are no published tensiometric CMC data or full surface tension isotherms for *A. amara* extracts [76]. This absence is notable given its long-standing hair-care use and high saponin content [62] and represents a primary methodological gap in assessing *A. amara* as a biosurfactant source [76].

Quantitative surfactant data are available for *A. procera*, and these provide the main point of reference within the genus. For *A. procera*, aqueous saponin extracts reduced the surface tension of water to approximately 46.6 ± 0.2 mN m^−1^ at a concentration of 6.0 × 10^−3^ g cm^−3^ at 25°C [80], with a CMC around 7.0 × 10^−3^ g cm^−3^ where marked changes in viscosity and conductivity indicated micelle formation [79,80]. A complementary dataset reports a reduced surface tension of approximately 43.8 mN m^−1^ at 1.0 × 10^−2^ g cm^−3^ at 20 ± 2°C for *A. procera* leaf saponins, again with CMC ≈7.0 × 10^−3^ g cm^−3^ [31,79]. The presence of CMC-dependent viscosity and conductivity transitions is characteristic of classical surfactant self-assembly behaviour and suggests that *A. procera* saponins undergo concentration-dependent structural rearrangement at the solution level [21,81].

More recent work on saponins from *A. procera* extracts reported moderate reduction in surface tension, low alkalinity, and measurable foaming and emulsifying capacity for the crude saponin extract [79,80]. In that study, maximum emulsion stability and an abrupt increase in solution viscosity and conductivity were again observed near the CMC, behaviour typical of classical surfactants at micellisation [81,82]. The dynamic interfacial properties of plant saponins at oil-water and air-water interfaces have been characterised using drop tensiometry and dilational rheology, revealing that saponin adsorption kinetics are controlled by a kinetically limited mechanism described by the Ward-Tordai model and that equilibrium interfacial pressure data follow the Gibbs adsorption isotherm [21,81]. Such detailed mechanistic studies are essential for understanding how natural saponins stabilise multiphase systems and form emulsions and foams [21,81].

When *A. procera* saponins are viewed alongside other plant saponins, their performance is intermediate [20,29]. Comparative data on multiple saponin sources indicate that saponins from *Acacia concinna* and *Genipa americana* can lower surface tension to approximately 32–34 mN m^−1^ at comparable or lower active concentrations [29,79], whereas *Sapindus mukorossi* and *Sapindus laurifolius* saponins typically reach approximately 35–39 mN m^−1^ at their CMC [83,84,85]. Conversely, *A. procera* saponins stabilise at approximately 44–47 mN m^−1^ in the reported systems [79,80], indicating clear surface activity but less pronounced tension reduction than the strongest plant-derived saponins currently documented [7,29]. Tea saponins (*Camellia oleifera*), for comparison, achieved CMC values of 0.5 g L^−1^ at 30 °C with surface tension reductions to 39.61 mN m^−1^ [29,79], while extracts of *Hedera helix* (ivy) saponins showed more variable performance depending on their degree of glycosylation, with hederasaponin B displaying a lower CMC than the more hydrophilic hederacoside C, and alpha-hederin (the aglycone) exhibiting the highest surface tension (≈39.8 mN m^−1^) among the three components tested [31,81]. Quinoa-derived saponins achieved a CMC of 1.2 g L^−1^ and reduced surface tension from 72.0 mN m^−1^ to 50.0 mN m^−1^ [86], while rhamnolipid biosurfactants demonstrated even more dramatic reductions to 26.40 mN m^−1^ and CMC values as low as 27.04 mg L^−1^ [87,88]. These comparative studies underscore the importance of structural features—particularly the number and composition of sugar residues and the lipophilic backbone—in determining surfactant efficiency [31,59,82].

Until recently, the only semi-quantitative performance metrics available for *A. amara* were the foaming index and crude saponin yield from the Rao shampoo-powder screening [76]. The first quantitative measurements on raw A. amara leaf powder suspensions now provide apparent surface tension values and basic emulsion performance metrics under simple model conditions [75], but there are still no CMC determinations, dynamic or equilibrium surface tension isotherms, systematic foam-stability measurements as a function of concentration, or quantitative emulsion stability measurements under fully standardised conditions. The absence of such data is particularly significant because comprehensive characterisation would require: (i) extraction and partial purification of saponin-rich fractions; (ii) measurement of equilibrium surface tension as a function of concentration to determine the CMC precisely using tensiometry; (iii) dynamic surface tension measurements using drop tensiometry or dynamic light scattering to assess adsorption kinetics [21,81]; (iv) quantitative foam stability testing following standardised protocols (such as the Bartsch or draining method) [79,89]; and (v) emulsifying capacity measurements across a range of oil types and concentrations [20,86]. At the genus level, detailed surfactant characterisation beyond *A. procera* is still sparse: reviews of *Albizia* phytochemistry and pharmacology focus predominantly on hemolysis, cytotoxicity and other bioactivities rather than classical interfacial metrics [43,62].

The methodological gap for *A. amara* represents a significant opportunity. Targeted tensiometric and rheological investigation would not only establish whether *A. amara* saponins perform within the range observed for other *Albizia* species—and hence whether they are suitable for cosmetic and food applications—but would also provide the quantitative basis necessary for direct comparison with established plant-derived and synthetic surfactants used in commercial shampoos and cleansing formulations [32,76]. Such data are prerequisites for formal product development and regulatory approval in cosmetic markets where claims of “natural” efficacy must be substantiated with rigorous physical and chemical evidence [7,32,77].

### 5.3. Comparative Performance and Limitations of the Current Evidence

The limited quantitative dataset allows only cautious comparison between *Albizia* saponins, other plant saponins and conventional synthetic surfactants. Using *A. procera* as a proxy, the reported CMC (~7 × 10^−3^ g cm^−3^) is substantially higher than that of sodium lauryl sulphate (SLS, ≈2 × 10^−3^ g cm^−3^) [32,79], and the minimum equilibrium surface tension (≈43–47 mN m^−1^) is higher than the ≈39 mN m^−1^ typically reported for SLS at CMC under similar conditions [79,80,90]. In purely tensiometric terms, the currently available *Albizia* saponin extracts therefore appear less efficient than SLS [32,80] and broadly comparable to mid-range plant saponins [29,83]. Recent comparative studies on plant-derived and microbial biosurfactants have established quantitative benchmarks: plant-based saponins reduced surface tension to 30–50 mN m^−1^ while rhamnolipid biosurfactants achieved 26.40–30 mN m^−1^ [32,86,87], confirming that *A. procera* performance falls within the intermediate range of natural surfactants.

Formulation-based comparisons are consistent with this quantitative picture but are challenging to interpret directly. In comparative evaluations of natural surfactant extracts, *Acacia concinna* saponins showed cleaning and foaming performance comparable to commercial baby shampoo under the specific test conditions, whereas *A. procera* saponins showed lower, but still measurable, detergency and foam formation [33,79,80]. Subsequent reviews summarising these and related data similarly classify *A. procera* as an effective but not high-performing natural surfactant when benchmarked against both synthetic and best in class plant saponins [29,79].

These comparisons underline two main limitations of the current evidence base. First, data across studies are difficult to compare directly because extraction methods, assay conditions (pH, ionic strength, temperature) and performance metrics (foam height vs. foam stability, emulsion type, oil phase) are not standardised [91,92,93]. Standardisation in surfactant characterisation is recognised as a critical challenge, with different laboratories employing different protocols that yield non-comparable results [93,94]. For example, variations in pH and ionic strength significantly influence CMC measurements and surface tension values [21,81], yet these parameters are often unreported in older literature on plant saponins. Temperature-dependent effects on foam stability and surface tension also complicate direct comparisons across studies [20,29]. Second, most available data for *Albizia* species concern crude extracts or complex formulations rather than defined, standardised saponin fractions [76,80], so the intrinsic efficiency of *Albizia* saponins themselves remains somewhat uncertain [16,93]. The use of impure preparations and poorly characterised congeners represents a significant methodological barrier to rigorous biosurfactant evaluation [93,94].

Within the broader plant-saponin landscape, *Albizia* saponins clearly exhibit surface activity and can support foaming and detergency in practical formulations [33,79,80], but based on current measurements they do not yet stand out as superior to the better-characterised plant saponins already explored for personal-care and household products [7,29]. From the perspective of this review, the key implication is not that *Albizia* saponins already match or outperform synthetic surfactants, but that (i) *A. procera* provides a genus-level proof-of-concept for surfactant behaviour [79,80], and (ii) *A. amara*, despite strong ethnocosmetic use and high saponin content [62,76], still lacks the basic interfacial characterisation (surface tension isotherms, CMC, foam and emulsion stability, and performance in model formulations) [21,76] needed to rigorously evaluate its potential as a biosurfactant source [93]. Systematic studies addressing these gaps are therefore a critical prerequisite for positioning *A. amara* alongside better-characterised plant saponins in cosmetic and food applications [7,14,95].

Taking a broader view, benchmark saponin-based surfactants such as *Quillaja saponaria* and *Sapindus* spp. Combine relatively low CMC values (typically in the 10^−4^–10^−3^ g cm^−3^ range) with equilibrium surface tensions in the ≈30–40 mN m^−1^ range under typical use conditions [32,85], and in the case of *Quillaja* are supported by well-defined compositional specifications and regulatory safety dossiers for food use [96]. By contrast, the best-characterised *Albizia* system (*A. procera*) exhibits higher CMCs (≈7 × 10^−3^ g cm^−3^) and stabilises at higher residual surface tensions (≈44–47 mN m^−1^ at 20–25 °C), indicating somewhat lower surfactant efficiency on a purely tensiometric basis [31]. On current evidence, therefore, *A. amara* and other *Albizia* species are unlikely to displace *Quillaja* as drop-in replacements in applications that demand maximal surface-tension reduction [97]. Their more plausible niche lies in regionally available, moderate efficiency saponin systems embedded in multifunctional extracts that also contribute antioxidant, conditioning or bioactive effects, particularly in cosmetic formulations where such combined properties are valued [76].

## 6. Cosmetic Applications of *Albizia amara* and Other *Albizia* Species

### 6.1. Traditional and Current Use of A. amara (“Arappu”) in Hair Care

Several ethnobotanical and pharmacognostic reports describe the use of *Albizia amara* leaves in South India as a powdered hair cleanser known locally as “arappu,” typically prepared by shade-drying and milling the foliage and applying the powder as a paste with water [62,96]. In these sources, *A. amara* leaf and flower preparations are mentioned for hair-related indications (hair fall, dandruff, general scalp care) in addition to their broader medicinal use for skin disorders and poisonous bites [43,62,96]. Within this corpus, arappu is repeatedly characterised as a plant-based shampoo or hair-wash powder rather than solely as a medicinal adjunct, implying that cleansing and conditioning functions are central to its traditional use [76,97].

Phytochemical screenings of *A. amara* leaves and bark consistently demonstrate the presence of saponins together with flavonoids, tannins and other polar metabolites [36,61,96], providing a plausible mechanistic basis for this cleansing role. Given the established surfactant behaviour of plant saponins in other species [6,79], it is reasonable though still inferential to attribute a significant part of the observed detergency and foaming behaviour of arappu to its saponin fraction [7,29], with co-extracted phenolics and flavonoids potentially contributing antioxidant or soothing effects on the scalp [6,36]. Traditional sources, however, do not quantify surfactant performance, and the link between reported benefits (e.g., reduced dandruff, perceived hair softness) and specific mechanisms remains largely qualitative [76].

### 6.2. Evidence from Formulated Products and Performance Tests

An early and influential evaluation of *A. amara* as a hair-care ingredient is the powder-shampoo study by Rao, who screened 21 herbal materials commonly used in Indian hair-care practices [76]. Under the extraction conditions employed, *A. amara* leaves showed the highest crude saponin yield among the plants tested (0.77 g) and a foaming index >100 across ten serial dilutions, indicating persistent foam formation in aqueous solution [76]. Polyherbal powder shampoos prepared with *A. amara* leaf powder and other herbs (e.g., *Acacia concinna*, *Sapindus mukorossi*, *Phyllanthus emblica*) were subsequently assessed for basic quality attributes [76]. In these formulations, pH values were within the slightly acidic range typical for scalp-care products, cylinder foaming tests indicated visually stable foam, and small panel evaluations reported satisfactory cleansing and ease of rinsing [76,98].

Since these formulations are multi-component systems, the specific contribution of *A. amara* cannot be deconvoluted from that of co-ingredients [76]. Nevertheless, these data illustrate that arappu-containing powder shampoos can be formulated to meet basic pH, foaming and user-assessed cleansing criteria without relying on synthetic surfactants in the solid phase [97,98]. Similar polyherbal powder and liquid shampoos (not always including *A. amara*) report comparable methodology and evaluation outcomes—slightly acidic pH, visually acceptable foam, and adequate cleaning scores—providing a benchmark for interpreting the *A. amara* results [99,100].

More recently, *A. amara* has been incorporated into an anti-dandruff shampoo formulation alongside Cynodon dactylon and sodium lauryl sulphate (SLS), where it is positioned primarily as an anti-dandruff and scalp-soothing agent [76,100]. In this liquid formulation, surface tension measurements of the finished shampoo (30.4–34.0 mN m^−1^ at 10% *w*/*v*) and cylinder foaming tests indicate performance within typical ranges for commercial shampoos [32]. Because SLS is present as the main surfactant, these data cannot be interpreted as direct evidence for the intrinsic surface activity of *A. amara* extracts; they do, however, demonstrate that *A. amara* powders and extracts can be compatibly integrated into liquid shampoos without compromising basic physicochemical properties [101].

In parallel, a broader cosmetic literature on plant saponins shows that standardised extracts from species such as *Sapindus mukorossi* [83,84], *Saponaria officinalis* [20,21], and *Camellia oleifera* [29,79] can reduce the surface tension of water to approximately 32–40 mN m^−1^, generate stable foam and provide detergency sufficient for shampoos, intimate soaps and facial cleansers, often allowing partial substitution of SLS/SLES [32,97]. These studies, while not *Albizia*-specific, define realistic performance targets for plant-derived saponins in cosmetic systems and contextualise the high foaming index and saponin content reported for *A. amara* leaves [7,29,79].

The bioactive benefits attributed to herbal shampoo formulations include antidandruff, antimicrobial, and anti-inflammatory properties, often demonstrated through incorporation of multiple plant extracts with complementary phytochemical profiles [100,102]. Clinical evaluations of polyherbal formulations have shown measurable reductions in dandruff, hair fall, and scalp irritation compared to untreated controls [102,103], though these benefits are typically attributed to the synergistic action of multiple ingredients rather than individual plant components [97,99].

Overall, the cosmetic evidence base for *A. amara* currently consists of: (i) consistent qualitative and semi-quantitative support for its inclusion in herbal powder and polyherbal hair-care products, and [76,98], and (ii) limited but increasing use in liquid shampoos, where its role is primarily as a bioactive adjunct rather than the sole surfactant [97,101]. However, the existing studies are methodologically modest: panel sizes are small, blinding and randomisation are rarely reported, and most formulations combine multiple plant extracts, making it impossible to attribute cleansing, antidandruff or conditioning benefits specifically to *A. amara*. In addition, none of these cosmetic applications include standardised tensiometric or interfacial characterisation of the *A. amara* fraction itself. As a result, while the traditional and herbal-shampoo literature strongly suggests that *A. amara* can contribute to acceptable hair-cleansing products, the quantitative contribution of its saponins relative to other plant and synthetic surfactants remains incompletely defined.

### 6.3. Alignment with Trends in Plant-Derived Saponins for Cosmetics

Recent reviews on surfactants in cosmetics highlight converging drivers for interest in plant-derived alternatives: concerns over irritation and environmental persistence of petroleum-derived anionics, regulatory and consumer pressure for biodegradable and renewable ingredients, and the appeal of multi-functional actives that combine cleansing with biological benefits [6,94,95]. Within this landscape, saponins are repeatedly identified as key plant-based candidates, owing to their amphiphilic structures, ability to reduce surface and interfacial tension, and capacity to stabilise foams and emulsions in personal-care formulations [6,7,14].

Several saponin-rich plants such as *Sapindus* spp. [83,84], *Quillaja saponaria* [20,21], and *Saponaria officinalis* [20,22] have already been developed into cosmetic ingredients and evaluated in shampoos, liquid cleansers and topical foams, sometimes with accompanying irritation/safety data [32,98]. In these systems, saponins typically function as primary or co-surfactants within mixed surfactant systems that may also include non-ionic or amphoteric components [101,104].

From a formulation and marketing perspective, *A. amara* shares several characteristics with these established saponin sources: it is a regionally abundant tree species [62,96], its leaves are already harvested and processed for a traditional hair-cleansing application [33,76], and its extracts contain saponins together with phenolic compounds that show antioxidant activity in preclinical models [6,36,62]. These features make *A. amara* a plausible candidate for development as a plant-derived surfactant for sulphate-free or “low-synthetic” hair and scalp products, particularly in markets where arappu use is culturally familiar [97,102].

At the same time, compared with better-studied saponin ingredients, *A. amara* currently lacks standardised extract specifications [76,93], cosmetic-focused irritation/sensitisation data [50,76], and comparative performance testing versus benchmark surfactants [32]—elements that are increasingly expected for cosmetic-grade raw materials [93,94]. The regulatory framework for cosmetic ingredients places significant emphasis on safety dossiers and standardisation [105,106], which remain substantial barriers to commercialization for under-characterised botanical materials [93,94].

Thus, while *A. amara* is aligned with several macro-trends in cosmetic surfactant development [6,94,95], its present status is best described as “promising but pre-commercial,” with a stronger foundation in traditional and pharmacological use than in formally characterised cosmetic performance [76,102].

### 6.4. Practical Formulation Considerations and Evidence Gaps

From the available data and the broader saponin literature, several practical formulation considerations and knowledge gaps emerge for *A. amara*. In terms of physical form and solubility, traditional arappu is used as a suspended leaf powder rather than as a dissolved surfactant [76,98], which is suitable for paste or powder shampoos but much less compatible with clear liquid systems [97,107]. Developing liquid or gel formulations centred on *A. amara* will therefore require saponin-rich extracts or fractions with defined solubility and reduced insoluble plant material [6,76,108].

Foaming indices reported for *A. amara* in powder-shampoo studies confirm that its leaf material contributes to measurable foam under standard in vitro tests [76], but these methods do not characterise foam structure (for example bubble size, “creaminess”) or its temporal stability during use [21,81]. Studies on other triterpenoid saponins suggest that they can produce relatively fine, stable foam [7,79], so *A. amara* saponins may offer similar sensorial potential, although this remains to be quantified directly [76,98].

Stability and compatibility represent a further gap. General saponin-focused reviews indicate that many plant saponins are stable over cosmetic-relevant pH and temperature ranges but can be sensitive to high ionic strength and certain electrolytes [7,37]. To date, however, no systematic study has mapped the stability of *A. amara* saponin fractions in the presence of typical cosmetic excipients such as oils, conditioning polymers, salts or fragrances [76], which is a practical barrier to their broader use in formulated products [50,109]. The stability of cosmetic formulations depends critically on compatibility between surfactants, emollients, and preservatives [110,111], a relationship that remains uncharacterised for *A. amara*.

Safety and regulatory aspects also require attention. Extracts of *A. amara* show antioxidant [36,62], antihyperlipidaemic, anticancer and anticonvulsant activities in preclinical models [43,62], reflecting the presence of flavonoids and macrocyclic alkaloids (e.g., budmunchiamines) in addition to saponins [62,96]. While these activities are of pharmacological interest, they underline the need for careful toxicological evaluation if *A. amara* extracts are to be used as cosmetic surfactants [112,113]. At present, there are no cosmetic-specific irritation or sensitisation studies on *A. amara* extracts, in contrast to some other saponin-rich ingredients that have undergone preliminary safety testing [50,114]. Modern cosmetic safety assessment typically employs in vitro and reconstructed tissue models to evaluate irritation and sensitization potential [50,115], methods that should be applied to *A. amara* extracts before regulatory submission.

In summary, existing evidence shows that *A. amara* leaf material can be incorporated into functional herbal powder shampoos [76,98] and SLS-containing anti-dandruff shampoos [76,100], and that its high saponin content is consistent with a potential role as a plant-derived surfactant in cosmetic formulations [6,76]. However, rigorous data on isolated *A. amara* saponins in modern cosmetic systems covering standardised surface-active measurements [32,91], long-term emulsion stability [50,109] and comprehensive safety profiling [105,114] are still largely lacking. Addressing these gaps will be essential if *A. amara* is to progress from a traditional arappu ingredient to a fully characterised, regulatory-compliant surfactant for contemporary hair- and skin-care products [76,93,94].

## 7. Prospective Food Applications of *Albizia*-Derived Saponins

### 7.1. Plant Saponins as Foaming and Emulsifying Agents in Foods

In contrast to their still-emerging role in cosmetics, certain plant saponins are already established as functional food ingredients [7]. The best-characterised example is *Quillaja saponaria* bark extract, which is authorised in multiple jurisdictions (e.g., as E 999 in the EU) [7,18] primarily as a foaming agent in soft drinks and frozen beverages, and as an emulsifier in beverage bases and flavour-oil concentrates [116]. Regulatory evaluations by JECFA and EFSA explicitly attribute these functions to the saponin fraction and define specifications, use levels and acceptable daily intakes [7], confirming that at least some plant saponin systems can meet both technological and safety requirements for food additives [18].

Recent colloid and food-science studies show that saponins can act as effective emulsifiers and foam stabilisers in model food systems when appropriately purified and formulated [117,118]. *Quillaja* saponins, for example, have been used to prepare oil-in-water nanoemulsions (e.g., thymol, oregano or other lipophilic actives) with droplet sizes typically in the tens to a few hundred nanometres [18], and with good kinetic stability over storage, moderate heating and changes in pH or ionic strength [117,119]. Comparative work on astaxanthin-enriched canola oil emulsions indicates that *Quillaja* and *Chenopodium quinoa* saponins can generate and stabilise submicron droplets with stability against pH 2–10 [118,120], 0–500 mM NaCl [117,119], and moderate thermal treatment [117,121], in some cases approaching the performance of Tween-type synthetic surfactants under the specific conditions tested [118].

Broader reviews of saponins in foods emphasise that these amphiphiles can contribute to foam stability (e.g., in beers and soft drinks) [7,18], emulsification [117,122], and possibly oxidative protection of lipids [123], but also stress that functionality depends strongly on plant source, degree of purification and formulation environment [7,124].

At the same time, food-focused reviews underline that saponins are not straight-forward drop-in replacements for synthetic surfactants [125]. Their interfacial behaviour is highly sensitive to pH [117,119], ionic strength [119] and co-solutes [117], and their technological advantages must be balanced against taste (bitterness, astringency) and potential antinutritional or bioactive effects [126,127]. In particular, several sensory and compositional studies in pulses and legumes identify saponins as contributors to bitter and astringent notes [126,128], which can become limiting when their concentrations approach those needed for strong interfacial effects [125,129].

Overall, the current state of the field shows that a small number of well-characterised saponin systems, above all *Quillaja*, are already accepted as food emulsifiers and foaming agents [7,18], whereas most other plant saponins remain at the stage of laboratory-scale feasibility studies [79,118].

### 7.2. How Albizia Saponins Relate to Food-Use Systems

Against this backdrop, *Albizia*-derived saponins are structurally compatible with known food-grade saponin systems but remain largely untested in true food matrices [76]. As summarised in Section 3 and Section 4, multiple *Albizia* species yield triterpenoid saponins based on oleanane- or echinocystic-type aglycones with multi-sugar chains [18,21]; these architectures are closely analogous to those reported for *Quillaja* and other food-relevant saponins [7,18]. From a surfactant perspective, such amphiphiles are expected to adsorb strongly at oil–water and air–water interfaces and to form micelles or other aggregates at concentrations in the low g L^−1^ range [21,22], consistent with behaviour observed for triterpenoid saponins generally [7,18].

Within the genus, quantitative interfacial data are available mainly for *A. procera* leaf extracts. These saponin-rich fractions reduce the surface tension of water to the mid-40 mN m^−1^ range at concentrations around 10^−2^ g cm^−3^ [80] and show changes in viscosity and conductivity near the CMC [80], together with moderate foaming and emulsifying capacity [43]. When placed alongside other plant saponins compiled in comparative tables of surface tension and CMC [18,21], *Albizia* extracts clearly exhibit surface activity [80] but do not reach the lowest surface tension values achievable with systems such as *Genipa americana* or some *Sapindus* preparations [83].

For *A. amara* itself, the evidence base is much thinner. Ethnocosmetic use as “arappu” hair-wash powder and the polyherbal shampoo study demonstrate high crude saponin content (0.77 g under their extraction conditions) [76] and a foaming index >100 over multiple dilutions [76], with acceptable cleansing and sensory performance in powder shampoos that blend *A. amara* with other saponin-rich plants [76]. More broadly, this review and related work show that *A. amara* leaf and bark extracts contain amphiphilic mixtures of saponins [62,96], flavonoids and other metabolites [36] capable of lowering surface tension [21] and stabilising emulsions under cosmetic-type conditions [117,122]. However, no studies have yet formulated *A. amara* extracts directly into food systems (e.g., beverages, dressings, aerated foods) [76], nor have they benchmarked *Albizia* saponins against authorised food saponins such as *Quillaja* under harmonised conditions of pH [117,119], ionic strength [119,130] and processing history [122].

On the basis of structure and the available surfactant data, it is therefore reasonable to regard *Albizia* saponins as potential candidates for food emulsification and foaming roles in principle [7,18]. At the same time, the absence of any published *Albizia* applications in foods [76], and the reliance on proxy data from *A. procera* and cosmetic-type systems [117,122], mean that their prospective status must be clearly distinguished from the established position of *Quillaja* [7,18] or *Yucca* saponins [131] in current food practice [122].

### 7.3. Opportunities and Constraints for Future Food Applications of A. amara

If suitably enriched and purified fractions of *A. amara* saponins can be obtained, their triterpenoid glycoside structures and demonstrated surface activity suggest several plausible food functions by analogy with other saponins [6,7]. In principle, *A. amara* saponins could be investigated as foam stabilisers in carbonated or semi-frozen beverages [7,132], as emulsifiers in beverage flavour oils and oil-in-water dressings [70,117], and as interfacial stabilisers in nutraceutical or flavour nanoemulsions where small droplet sizes and kinetic stability are required [18,133,134]. A potential added benefit, compared with more narrowly purified saponin systems, is the co-occurrence of antioxidant flavonoids and phenolics in *A. amara* leaves and seeds [36]; if retained in a controlled way, these could contribute to oxidative stability of lipid phases in addition to interfacial stabilisation [123,135].

However, several constraints must be addressed before *A. amara* can be considered a realistic food-ingredient candidate. First, sensory impact is a major unknown. Saponins from legumes and other plants are frequently implicated in bitter and astringent notes [125,126,129], and targeted studies show that at least some individual saponins are clearly bitter at technologically relevant concentrations [129,136]. No data currently exist on the flavour thresholds or sensory profiles of *A. amara* saponin fractions [76], so there is a real possibility that the concentrations needed for robust foam or emulsion stabilisation may be organoleptically unacceptable without taste-masking or further purification [137,138].

Second, safety and bioactivity require particularly careful evaluation. As reviewed earlier, *Albizia* species often yield haemolytic and cytotoxic saponins [7,38], and *A. amara* seeds contain macrocyclic budmunchiamine alkaloids with potent DNA-interactive and cytotoxic activities [38]. While these alkaloids are not themselves surfactants, their co-extraction into crude or partially purified fractions would be incompatible with food use [38]. Any prospective food-grade *A. amara* ingredient would therefore need to demonstrate, via appropriate purification and toxicological testing, that haemolytic effects, cytotoxicity and other adverse activities are absent at intended use levels [7,27], in line with the safety dossiers that underpinned approvals of *Quillaja* extracts [7,18].

Third, *A. amara* currently has no recognised status as a food additive or novel food in major regulatory frameworks, unlike *Quillaja* [7,18] and some *Yucca* preparations [131,139] which appear in additive lists with defined specifications and permitted uses [7,131]. This implies that even if technical performance and safety were demonstrated, a full regulatory approval process would still be required [7]. Finally, there is at present no information on the behaviour of *A. amara* saponins in realistic food matrices under processing conditions involving heat treatment [140], wide pH ranges [140,141] and high ionic strengths [140,141], despite evidence from other saponins that phase behaviour and stability can change markedly outside relatively narrow windows [117,142,143].

In summary, the available literature supports the view that *Albizia*-derived saponins, including those from *A. amara*, possess the structural motifs and baseline surface activity required of food foaming and emulsifying agents [6,7]. Their practical use in foods, however, remains entirely prospective at this stage [76]. The most constructive path forward is a staged research programme that (i) isolates and structurally characterises *A. amara* saponin fractions [96]; (ii) quantifies their interfacial behaviour and emulsifying/foaming performance in model food systems [43,80]; (iii) establishes toxicological and sensory thresholds [38,125]; and (iv) benchmarks them against authorised saponin ingredients such as *Quillaja* [7,18] in standardised comparative studies [144,145]. Only once such evidence is available would it be possible to make evidence-based claims about *A. amara* as a food-grade biosurfactant, complementing its better-documented potential in cosmetic applications [95,146]. Taken together, these constraints mean that all proposed food uses of *A. amara* saponins must at present be regarded as speculative. No studies have yet examined *A. amara* fractions in real food matrices, established sensory acceptance thresholds, or generated the toxicological data that underpin approvals for existing food saponins such as *Quillaja* extracts. Until such evidence is available, *A. amara* is better viewed as a promising candidate for exploratory research rather than a near-term food emulsifier or foaming agent.

## 8. Toxicity, Safety and Regulatory Considerations

### 8.1. General Toxicity Profile of Plant Saponins

Plant saponins are amphiphilic glycosides with a well-documented capacity to interact with biological membranes [65,147]. Classical toxicological work and subsequent reviews show that many saponins cause dose-dependent haemolysis of erythrocytes in vitro and in vivo [24], particularly when administered parenterally, largely through complexation with cholesterol and disruption of lipid bilayers [65,147]. Orally, saponins can increase gastrointestinal epithelial permeability [148], induce local irritation and affect nutrient absorption [149]; they are often discussed as antinutritional factors in monogastric animals [150,151], although they have also been associated with potentially beneficial actions such as modulation of bile acid metabolism and serum cholesterol [24,152].

A characteristic feature of many saponin-containing extracts is marked toxicity to fish and certain aquatic invertebrates [24]. Reviews such as that by Francis et al. collate numerous examples in which saponin-rich seeds, leaves or crude extracts cause rapid death of fish at relatively low concentrations, primarily via damage to gill and respiratory epithelia [24]; this property underlies the traditional use of some saponin plants as fish poisons. Purified seed saponins from various species can exhibit low intraperitoneal LD_50_ values in rodents (often in the single-digit mg kg^−1^ range), with acute signs such as dyspnoea, ataxia and convulsions [153], again consistent with potent membrane activity at systemic exposures.

Early food-oriented reviews, written in the context of naturally occurring saponins in legumes and cereals, described dietary saponins as “practically non-toxic” to humans at usual intake levels, largely because they occur in complex matrices, are partly degraded or unabsorbed in the gut, and are ingested at relatively low doses [154]. More recent evaluations by JECFA and EFSA for saponin-containing materials used as food additives (e.g., *quillaia* extracts, mahua kernel cake) take a more explicitly risk-based approach [155,156], emphasising haemolytic potential, gastrointestinal irritation and species differences, and derive acceptable daily intakes from animal studies with adequate safety factors [155]. Taken together, these sources indicate that plant saponins can be tolerated orally at low exposures and may confer physiological benefits in some contexts [151], but that concentrated saponin preparations or non-oral routes readily produce haemolysis and systemic toxicity [65,153]. Route of administration, degree of purification and species sensitivity are therefore critical determinants of risk [155,156].

### 8.2. Toxicity and Safety of Albizia Species and A. amara

Within this general framework, *Albizia* species combine a long record of ethno-medicinal use with experimental evidence for high-potency constituents [157,158,159]. Ethnopharmacological surveys and traditional medicine systems document the use of A. julibrissin, A. lebbeck and related species for a variety of conditions including insomnia, mood disorders, respiratory complaints, skin diseases and parasitic infections in Chinese, Indian and African systems [159,160,161], typically as aqueous or hydroalcoholic preparations of bark, leaves or flowers, and these sources do not highlight acute toxicity at customary doses [158,159]. At the same time, many isolated *Albizia* saponins and alkaloids display pronounced cytotoxicity and other potent bioactivities in vitro and in animal models [67,162,163].

For *A. julibrissin*, crude saponin fractions and purified julibrosides show cytotoxic effects against a range of tumour cell lines [162]. Total saponins from *A. julibrissin* (TSAJ) demonstrated significant inhibition of VEGF-mediated angiogenesis in vitro and in vivo, modulating suppression of phosphorylated focal adhesion kinase, Akt, and extracellular signal-regulated kinase in the VEGF/VEGFR2 signalling pathway [162]. For *A. lebbeck*, a recent pharmacological overview catalogues antiallergic, anticancer, anticonvulsant and antiparasitic activities for various fractions [160,161]. The crude extract, fractions, and bioactive compounds exhibited potent activities against multiple targets [160,161], but the literature notes that many of these extracts and isolated saponins are cytotoxic at concentrations only modestly above their “active” range. Multiple pharmacological activities have been documented, including antidiabetic, antidiarrheal, anti-inflammatory, antimicrobial, antinociceptive, antioxidant, antipyretic, antivenom, estrogenic, neuroprotective, and nootropic effects [160,161], yet comprehensive toxicological characterisation remains limited and further studies are required to estimate potential side effects [160,161].

Separate toxicological accounts report livestock poisoning associated with certain plant species [164], thought to involve neurotoxic constituents, although in many cases the precise species and toxins are not fully characterised. These examples indicate that *Albizia* metabolites can have narrow experimental safety margins, but such effects have not been reported specifically for *A. amara* in ethnomedicinal contexts [158].

For *A. amara*, two groups of constituents are most relevant to safety: macrocyclic spermine alkaloids (budmunchiamines) and triterpenoid saponins. As detailed in Section 4, budmunchiamines A–C were originally isolated from *A. amara* seeds using DNA-binding assays, with later work extending the series to budmunchiamines D–I [38]; these macrocyclic alkaloids show strong interaction with calf thymus DNA and inhibit mammalian DNA and RNA polymerases and HIV-1 reverse transcriptase [38], with broad cytotoxic and antimicrobial activity in vitro. Importantly, these compounds have so far been characterised from seeds rather than leaves, and their presence in leaf-only preparations (such as traditional arappu powders) has not been systematically evaluated. From a safety standpoint, they represent a seed-associated hazard that must be excluded or controlled in any whole-plant or mixed-material extract intended for cosmetic or food use [38].

Several studies on *A. amara* leaf extracts report in vitro cytotoxic activity against human cancer cell lines. For instance, aqueous, ethanolic and ethyl-acetate leaf fractions have shown growth inhibition of MCF-7 breast cancer cells with IC_50_ values in the tens of µg mL^−1^ range after 24–48 h exposure, indicating that crude extracts contain constituents capable of affecting mammalian cell viability at relatively low concentrations. Other work documents antioxidant, hepatoprotective, antihyperlipidaemic and anti-inflammatory effects of *A. amara* extracts in rodent models, often without overt signs of acute toxicity at the doses tested, but comprehensive toxicological characterisation (e.g., genotoxicity, reproductive toxicity, long-term exposure) remains sparse [7]. In addition, investigations of saponin-rich *Albizia* seed extracts from Madagascan species have shown marked toxicity to fish and small animals [22], reinforcing the general concern that concentrated *Albizia* saponins and co-extracted metabolites may have limited safety margins in non-traditional exposure scenarios [7].

Two broad conclusions emerge for *A. amara*. The ethnomedicinal record for topical and oral preparations suggests that, when used in traditional forms and doses, *A. amara* can be administered without obvious acute toxicity. At the same time, the presence of highly bioactive seed alkaloids [38] and the cytotoxicity of certain leaf extracts in vitro indicate that the plant contains constituents with significant hazard potential, particularly when concentrated or reformulated [38]. Any surfactant or emulsifier fraction derived from *A. amara* must therefore be characterised not only for its saponin content but also for the removal or control of budmunchiamines [38] and other strongly bioactive components [38].

### 8.3. Implications for Cosmetic Versus Food Use

These data have different implications for cosmetic and food applications of *A. amara*-derived surfactants. In cosmetic products, the principal exposure route is dermal, and for shampoos and cleansers the contact is brief and followed by rinsing. Under such conditions, systemic absorption of high-molecular-weight, polar saponins is generally considered to be low, based on dermal-absorption data for analogous surfactants and the barrier function of intact stratum corneum [6,18], although this has not been specifically quantified for *A. amara* fractions. Cosmetic safety assessment therefore focuses on local effects (skin and eye irritation, barrier disruption, sensitisation) and on systemic exposure estimates derived from reasonably conservative assumptions [11]. Experience with other botanical surfactants suggests that many can be formulated safely in rinse-off systems at modest concentrations when pH and co-surfactants are appropriately controlled [94,157]. For *A. amara*, prudent development of cosmetic surfactants would require that surfactant fractions are derived from leaves (or other non-seed tissues), that seed-derived budmunchiamines are analytically demonstrated to be absent or below relevant limits of concern, and that at least in vitro skin/eye irritation tests (and, for leave-on products, sensitisation assays) are conducted on the intended ingredient [11,157].

For food applications, the requirements are considerably more stringent. Oral exposure entails direct contact with gastrointestinal epithelia and the possibility of systemic absorption, and safety assessment must address acute and repeated-dose toxicity, haemolytic potential, gastrointestinal effects, and possible genotoxicity and reproductive toxicity [7,157]. *Quillaia* extracts, the best-established food-grade saponin systems, have been evaluated by JECFA and EFSA as food additives (E 999) on the basis of subchronic and chronic toxicity studies, genotoxicity assays and haemolysis data [155], leading to acceptable daily intakes that explicitly account for these endpoints [155,156]. In the case of *A. amara*, there are currently no subchronic or chronic oral toxicity studies, no genotoxicity or reproductive-toxicity datasets, and no haemolysis thresholds for its specific saponin fractions [7]. Given the documented DNA-interactive budmunchiamines in seeds and the cytotoxic saponin fractions in congeners [38], it would be inappropriate to infer oral safety from traditional cosmetic use or from the safety record of other food saponins [24]. Any proposal to use *A. amara*-derived surfactants in foods would require a de novo toxicological programme and careful characterisation of the test material [156,158].

### 8.4. Regulatory Pathways for Plant-Derived Surfactants

Regulatory frameworks mirror these differences in exposure and evidence requirements. In cosmetics, for example, under EU Regulation 1223/2009, plant-derived surfactants such as saponin-rich extracts are treated as cosmetic ingredients that must be safe under reasonably foreseeable conditions of use [11]. They are not individually “approved” by authorities in the way food additives are; instead, the “responsible person” must compile a Cosmetic Product Safety Report, including toxicological profiles, exposure assessments and impurity information [11,94], and ensure that no ingredient contravenes specific restrictions in the regulation. For an *A. amara*–based surfactant, this would imply providing robust data on composition (including control of budmunchiamines and other alkaloids), skin and eye irritation, sensitisation (where relevant), and systemic exposure estimates under intended use [11].

In foods, surfactants intended as emulsifiers or foaming agents fall under food additive or novel food legislation, depending on jurisdiction and history of use [158]. *Quillaia* extracts again provide the clearest precedent: JECFA and EFSA opinions specify compositional criteria (e.g., saponin content, solvent residues) [155], evaluate toxicology and derive ADIs [155,156], and the EU additive list defines permitted uses and maximum levels for E 999 [156]. A new saponin-based ingredient without a recognised history of safe food use, such as an *A. amara* extract, would generally be treated as a novel food or new food additive [158] and would require a comprehensive dossier covering identity, manufacturing, proposed uses, anticipated intake, and a full toxicological data package [155,158].

In summary, plant saponins as a class can be used safely in cosmetics and foods at carefully controlled exposure levels [7,18], but their amphiphilicity and membrane activity necessitate case-specific assessment [7]. For *Albizia* species and *A. amara* in particular, the coexistence of empirically safe traditional uses with potent cytotoxic and DNA-interactive constituents [38] calls for a cautious, data-driven approach. Within cosmetics, reasonably purified *A. amara* saponin fractions derived from non-seed tissues could in principle be evaluated within existing cosmetic-safety frameworks [11], provided that budmunchiamines and other strongly bioactive co-constituents are adequately controlled [38] and local effects are characterised [11]. For food applications, by contrast, the current absence of targeted toxicological studies and regulatory evaluations means that *A. amara* must be considered a promising but unproven biosurfactant source [7] whose safety profile cannot be extrapolated from other saponins [24] and would need to be established de novo [156].

## 9. Research Gaps and Future Directions for *Albizia amara* as a Biosurfactant Source

The available literature establishes *Albizia* spp. as a saponin-rich genus and *A. amara* as a traditional cleansing agent with demonstrable bioactivity [38,159], but the dataset is still fragmentary when evaluated from a surfactant-science and application-oriented perspective. As discussed earlier, quantitative interfacial characterisation within the genus is largely confined to *A. procera*, where leaf extracts reduce surface tension into the mid-40 mN m^−1^ range at concentrations around 10^−2^ g cm^−3^ and show typical signatures of micellisation [80]. For *A. amara*, by contrast, robust performance metrics remain scarce: beyond crude foaming indices and qualitative emulsifying behaviour in cosmetic-type systems, only one recent study has quantified the apparent surface tension and emulsifying behaviour of raw leaf powder suspensions in simple aqueous systems [75]. There are still no surface tension isotherms, CMC values, or systematic foam/emulsion-stability data for defined saponin-rich extracts under well-defined conditions. Robust tensiometric and colloidal characterisation of *A. amara* saponin-rich fractions, generated using standardised protocols [9,18], is therefore a primary requirement if its surfactant potential is to be meaningfully compared with other plant saponins [9,85] and synthetic benchmarks [4].

A second gap concerns extraction and fractionation strategies optimised specifically for surfactant use. Existing *A. amara* studies have generally employed broad solvent extractions (aqueous, hydroalcoholic, methanolic) designed for pharmacological screening rather than for generating defined surfactant fractions. There is currently no systematic work on how extraction solvent, pH, ionic strength, solid–liquid ratio, or post-processing steps (e.g., precipitation, membrane concentration, chromatography) influence saponin yield, purity, and interfacial performance [160]. By analogy with work on *Quillaja* and *Sapindus* [18,83], targeted process development could produce *A. amara* fractions that balance surfactant efficiency with acceptable sensory and safety profiles, but this remains unexplored. Response surface methodology and optimisation approaches, as demonstrated for other saponin-rich plants [29,161], could systematically define the extraction parameters that maximise surfactant-relevant properties whilst minimising cytotoxic co-constituents [38].

Third, formulation-level evidence for *A. amara* is confined to herbal powder shampoos and a small number of model emulsions under cosmetic-relevant conditions. There are no controlled comparative studies that embed *A. amara* fractions in standardised cosmetic or food matrices, benchmark them against well-characterised saponins [7,18] and synthetic surfactants [14] on an active-matter basis, and assess not only foaming and detergency but also rheology, sensorial attributes and long-term stability [9]. Without such work, it is not possible to judge whether *A. amara* is best positioned as a primary surfactant, a co-surfactant [22], or a niche multifunctional additive [8,162].

A fourth, critical gap lies in toxicology and ecotoxicity. While budmunchiamine alkaloids and cytotoxic saponin fractions from *Albizia* spp. have been characterised in vitro [38,161], and fish toxicity is well documented for other saponin-rich seeds [24], there are no dedicated safety studies on *A. amara* surfactant fractions: no haemolysis thresholds [26], no dermal-irritation or sensitisation data, no subchronic oral studies and no ecotoxicity data in aquatic organisms under environmentally realistic exposures [164]. For cosmetic applications, this limits the ability to perform robust margin-of-safety evaluations [11]; for proposed food use, it precludes any serious regulatory consideration [155,156].

Finally, techno-economic and sustainability dimensions have yet to be addressed. *A. amara* is widely distributed in dryland regions and is used locally as arappu, but its potential availability as an industrial feedstock, the costs and energy intensity of producing saponin-rich fractions, and its performance relative to competing natural surfactants (e.g., *Quillaja*, *Yucca*, *Sapindus*) [18,83,85] have not been quantified. Life-cycle assessments [4] comparing *A. amara*–based surfactants with conventional petrochemical surfactants [13] and with other botanical saponins [7] would be needed to substantiate any claims of environmental advantage [8,165]. These gaps define a clear research agenda: rigorous interfacial measurements [9,80], process optimisation [29,161], application-focused formulation studies [162], comprehensive safety assessment [155,164] and integrated techno-economic/sustainability analyses [4,13].

## 10. Conclusions

Plant-derived saponins are attracting sustained interest as candidates for safer, more sustainable surfactants in cosmetics and foods. Within this broader landscape, the genus *Albizia* stands out for its richness in triterpenoid saponins, its long history of medicinal use, and its demonstrated surface activity in species such as *A. procera*. *Albizia amara* occupies a distinctive position in this group. Ethnocosmetic practice in South India treats its leaf powder (arappu) as a practical hair cleanser and conditioner; phytochemical surveys confirm the presence of saponins alongside flavonoids and other amphiphiles; and emerging formulation work shows that *A. amara* extracts can contribute to foaming and emulsification in cosmetic-type systems.

In contrast to benchmark systems such as *Quillaja saponaria* saponins, which benefit from fully elucidated structures, quantitative CMC and surface tension data, established food uses and regulatory evaluations, *A. amara* remains under-characterised from a surfactant-science and safety perspective. Its saponin structures are largely inferred rather than directly determined, and although recent baseline measurements on raw *A. amara* leaf powder suspensions have provided the first quantitative indications of apparent surface tension reduction and emulsifying behaviour, robust interfacial metrics for defined saponin-rich extracts are still missing; formulation studies remain limited and context-specific, and dedicated toxicological work on surfactant fractions is lacking. The presence of highly bioactive budmunchiamine alkaloids in the seeds and cytotoxic fractions in leaves further underscores that safety cannot be assumed from traditional use or from analogy with other saponin sources.

Despite these deficiencies, *A. amara* is a particularly rational focus among underexplored saponin plants for several reasons. Unlike many “novel” candidates identified purely from screening, it already has a well-established role as a cleansing agent in a large, long-standing user population, providing a rare combination of empirical performance evidence and practical know-how around harvesting and processing. Its distribution in dry, resource-constrained regions and use as a locally available hair-wash suggest that, if suitable fractions can be developed, *A. amara* could support regionally grounded, plant-based surfactant systems rather than relying solely on globally traded tree barks or imported seeds. At the same time, the coexistence of saponins with antioxidant flavonoids and other bioactives offers the prospect, if carefully controlled, of multifunctional ingredients that combine interfacial activity with additional protective effects.

## Data Availability

No new data were created or analyzed in this study. Data sharing is not applicable to this article.
